# Editorial: Legumes and their microbiome in climate change mitigation

**DOI:** 10.3389/fpls.2023.1220535

**Published:** 2023-06-12

**Authors:** Mariela I. Monteoliva, Oscar A. Ruiz, Fadong Li

**Affiliations:** ^1^ Instituto de Fisiología y Recursos genéticos Vegetales, Unidad de Estudios Agropecuarios (IFRGV-UDEA), Instituto Nacional de Tecnología Agropecuaria (INTA), Concejo Nacional de Investigaciones Científicas y Técnicas (CONICET), Córdoba, Argentina; ^2^ Facultad de Ciencias Agropecuarias, Universidad Católica de Córdoba (UCC), Córdoba, Argentina; ^3^ Universidad Nacional de San Martín (UNSAM), San Martín, Argentina; ^4^ Instituto Tecnológico de Chascomús (INTECH), CONICET, Chascomús, Argentina; ^5^ Key Laboratory of Ecosystem Network Observation and Modeling, Institute of Geographic Sciences and Natural Resources Research, Chinese Academy of Sciences (CAS), Beijing, China; ^6^ College of Resources and Environment, University of Chinese Academy of Sciences, Beijing, China

**Keywords:** plant-beneficial microorganisms, Fabaceae, sustainable agriculture, environmental stress, pulses

Climate change accelerates or enhances the incidence of a variety of abiotic and biotic stresses on agricultural and forest productivity. By changing different aspects of agriculture management, we can lower the risk of climate change over plant-derived productivity, including food, fiber, and bioenergy.

Legumes (Fabaceae) is the third largest family of flowering plants, with more than 20000 species. They play central roles in (human and animal) food and plant-based protein production. Legume crops emit fewer greenhouse gases (such as carbon dioxide and nitrous oxide, compared to other nitrogen-fertilized crops), allow more sequestration of carbon in soils, and save fossil energy inputs in the system by reducing N fertilizer needs. In intercropping systems, legumes may facilitate circulation of soil nutrients (such as N, P, Fe, and Zn), water retention and availability, and breaking pest and disease cycles, depending on the climate, soil, and management of agro-ecosystems in which they are included (Stagnari et al., 2017).

Mutualistic relationships of legumes with either bacteria or fungi have been investigated intensively, usually as bi-partite interactions. Their collective contribution to plant growth and performance appears to be affected by crop genotype, microbial interactions, and environmental variables. However, diverse symbiotic and mutualistic interactions take place simultaneously or sequentially under field conditions (Tsiknia et al., 2021). The interaction with the microbiome, as well as the outcome of microbe-microbe interactions, enable plants to receive a wide range of benefits like increased tolerance against biotic and abiotic stresses, biological control of pathogens, and enhanced nutrient acquisition and growth (reviewed by Monteoliva et al., 2022). It is now clear that plants’ adaptability to changing environments depends not only on their phenotypic plasticity but also on their ability to recruit, establish or shift their microbiome (Uroz et al., 2019).

Overall, the legume holobiome (legume and its microbiome as a unit) is a promising tool to mitigate the climate change impact while addressing the need for healthy and nutritious food for a rapidly growing population. The challenge is to understand the dynamics of the legume-intercrop-microbiome-soil system and use those insights as a solid basis for developing new integrated technologies and practices. In addition, with the rapid development of the bioinput market, a comprehensive evaluation of the impact of the exogenously introduced microbes on the native microbiota is urgently needed.

This Frontiers in Plant Science virtual Research Topic *on Legumes and their Microbiome in Climate Change Mitigation* consists of 4 publications: 2 original research articles and 2 reviews, on three main topics: breeding, intercropping, and microbiome. One of the articles reviews the relevance of studying and improving underutilized legumes, such as Bambara groundnut, considering them as an holobiome. Another article reports how soil water availability affects the competitiveness of a co-dominant legume *Lespedeza davurica* in an intercropping system, and the last two articles focus on the effect of beneficial bacteria, studying how the beneficial bacteria *Pseudomonas monteilii* could protect the forest plantation of *Dalbergia sissoo* from mortality caused by fungi, and the effect of rhizobia inoculation and P fertilizer increasing yield of three grain-legumes in a meta-analysis.

As summarized below, each paper ties legume crops and their microbiome with some climate change-derived challenges.

Breeding programs have been largely developed in plants’ optimal growing conditions, losing some relevant genes for the interaction of the crops with beneficial microorganisms (Marco et al., 2022). Ajilogba et al. highlight the agronomic and nutritional advantages of Bambara groundnut (*Vigna subterranea*) which is the third most-eaten legume in sub-Sahara Africa. It has wild and cultivated varieties with a high ability to tolerate drought, grown in marginal soils, and large genetic variability to improve the crop. The authors emphasized the need to improve this type of underutilized legumes preserving the interaction with their microbiomes, which must complement the use of the major crops shortly in the context of climate change.

Intercropping is a good management tool to improve yields by combining mutual benefits for different species (such as grasses and legumes). Xu et al. studied the effect of soil water availability in the intercropping system formed by the two native forage species from China: the legume *Lespedeza davurica* and the grass *Bothriochloa ischaemum.* The higher water availability produced a higher increase in the herbaceous grass biomass when in mixtures, indicating that the legume presence improved the yield of the grass. The more stable ratio of both species in a mixture was obtained at 10:2 (*B. ischaemun: L davurica*) when their intra- and interspecific competitiveness tended to be equal, providing the highest water use efficiency and total relative yield.

Considering the microbiome of legumes in further research is critical to improving the yield and quality of legume production, as much as crop protection. Buernor et al. carried out an interesting meta-analysis over 33 publications conducted in Ghana, to review what is known about the effect of biological nitrogen fixers (BNF) and P fertilization on the yield of 3 legumes (groundnut, soybean, and cowpea) in four agroecological zones of the region. The combined treatment with BNF and P fertilizer was the major yield driver in soybean, while genotype was the first factor for cowpea and peanut. Although in the three crops, the combined fertilization treatment was the most effective to increase yield, the highest average yield change was observed in cowpea. Results suggest that breeding programs and BNF inoculation technologies for groundnut and cowpea could largely benefit low-income farmers in Ghana, in comparison to soybean which present already good elite genotypes and effective inoculants.

On the other hand, Srivastava et al. characterized the native bacteria, *Pseudomonas monteilii* MN759447, previously isolated from Shisham (*Dalbergia sissoo*) forest ecosystem in the Western Himalayas. They aim to study a biocontrol agent for the dieback and wilt of the forest. The causal agent of the high mortality rates is unknow, but the pathogenic fungi *Aspergillus calidoustus*, *Talaromyces verruculosus*, *Fusarium oxysporum*, and *Talaromyces pinophilus* are present in the forest and *P. monteilii* showed antifungal effect against them. The strain was positive for siderophore production, and two pseudomonine derivatives were identified by LC-MS, supporting a potential use of the bacterial strain as a commercial product to improve the health and nutrition of the forest.

## Author contributions

MI, RA, and FL draft revised and approved the editorial text. All authors contributed to the article and approved the submitted version.
